# Synthesis, crystal structure and Hirshfeld surface analysis of bis­{2-[(pyridin-2-yl)amino]­pyridinium} tetra­cyano­nickelate(II)

**DOI:** 10.1107/S205698902001419X

**Published:** 2020-10-30

**Authors:** Zouaoui Setifi, Hela Ferjani, Fatima Setifi, Safa Ezzine, Mohammed Hadi Al-Douh

**Affiliations:** a Laboratoire de Chimie, Ingénierie Moléculaire et Nanostructures (LCIMN), Université Ferhat Abbas Sétif 1, Sétif 19000, Algeria; b Département de Technologie, Faculté de Technologie, Université 20 Août 1955-Skikda, BP 26, Route d’El-Hadaiek, Skikda 21000, Algeria; c Chemistry Department, College of Science, IMSIU (Imam Mohammad Ibn Saud Islamic University), Riyadh 11623, Kingdom of Saudi Arabia; d Department of Chemistry, College of Sciences, King Khalid University, Abha, Saudi Arabia; e Chemistry Department, Faculty of Science, Hadhramout University, Mukalla, Hadhramout, Yemen

**Keywords:** crystal structure, tetra­cyano­nickelate, *N*-(pyridin-2-yl)pyridinium-2-amine, hydrogen bonding, Hirshfeld surface analysis, crystal structure

## Abstract

In the title mol­ecular salt, (C_10_H_10_N_3_)_2_[Ni(CN)_4_], the dihedral angle between the pyridine rings in the cation is 1.92 (13)° and the complete anion is generated by a crystallographic centre of symmetry. An intra­molecular N—H⋯N hydrogen bond occurs in the cation, which closes an *S*(6) ring. In the crystal, the components are linked by N—H⋯N and weak C—H⋯N hydrogen bonds, which generate chains propagating in the [101] direction. Weak aromatic π–π stacking inter­actions are also observed. A Hirshfeld surface analysis and two-dimensional fingerprint plots indicate that the most important contact types in the crystal packing are N⋯H/H⋯N, C⋯H/H⋯C and H⋯H with contributions of 37.2, 28.3 and 21.9%, respectively.

## Abstract 2

The title structure consists of [Ni(CN)_4_]^2–^ square-planar anions separated by layers of (C_10_H_10_N_3_)^+^ cations. The crystal packing features N—H⋯N hydrogen bonds, which generate [101] chains.

## Chemical context   

Transition-metal coordination compounds, where CN^−^ ligands play the main structure-forming role, so-called cyano­carbanion or cyano­metallate complexes, have been the subject of inter­est for many years, in particular due to their magnetic properties (Ferlay *et al.*, 1995 ▸; Bretosh *et al.*, 2020 ▸; Benmansour *et al.*, 2012 ▸; Setifi *et al.*, 2009 ▸; Yuste *et al.*, 2009 ▸; Addala *et al.*, 2015 ▸), including spin-crossover behavior (Benmansour *et al.*, 2010 ▸; Yoon *et al.*, 2011 ▸). The square-planar tetra­cyano­nickelate(II) anion [Ni(CN)_4_]^2–^ has proved to be very versatile and diverse in both coordination chemistry and magnetism.
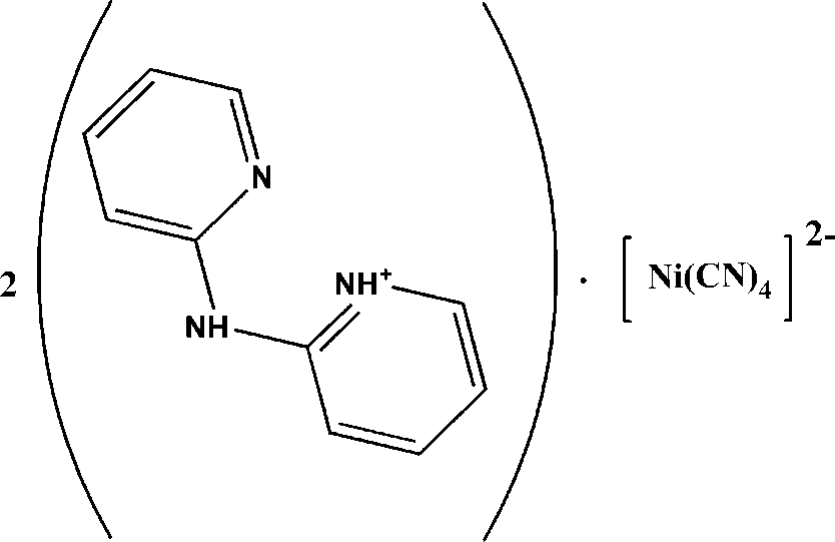


We have been inter­ested in using the tetra­cyano­nickelate(II) anion in combination with other chelating or bridging neutral co-ligands to explore their structural features and properties relevant to the field of mol­ecular materials exhibiting the spin-crossover phenomenon (Setifi *et al.*, 2013 ▸, 2014 ▸; Kucheriv *et al.*, 2016 ▸). During the course of attempts to prepare such complexes with 2,2′-di­pyridyl­amine (dpa), we isolated the title mol­ecular salt, (I), whose mol­ecular and supra­molecular structure is described herein.

## Structural commentary   

**Figure 1:**
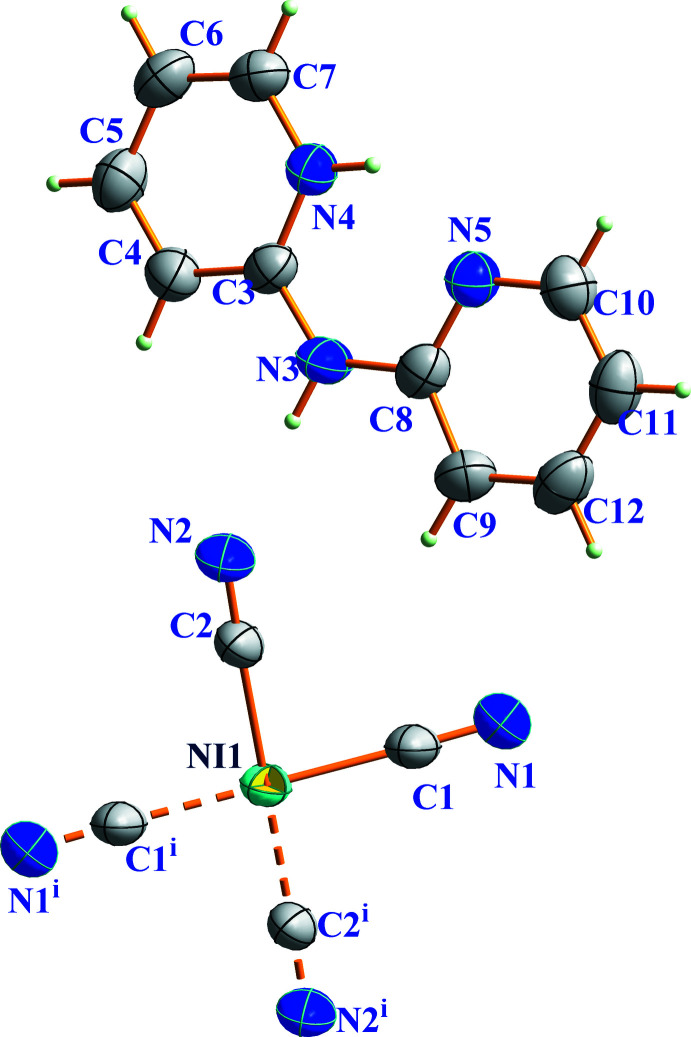
The mol­ecular structure of (I) with displacement ellipsoids drawn at the 50% probability level. Symmetry code: (i) −*x* + 1, −*y*, −*z*

**Figure 2:**
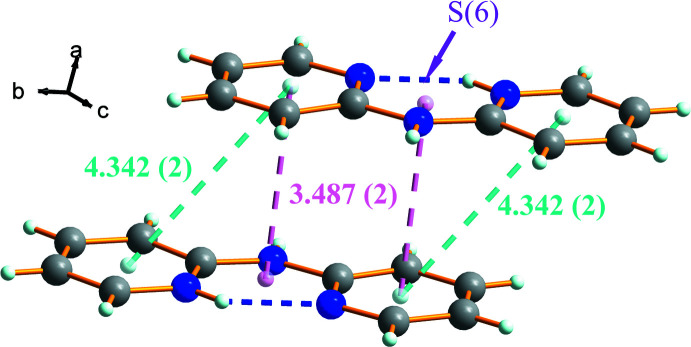
Offset and parallel π–π-stacking inter­actions (broken lines) in the cation–cation chains.

The asymmetric unit of (I) contains one (C_10_H_10_N_3_)^+^ cation and one half of a [Ni(CN)_4_]^2−^ anion (Fig. 1 ▸). The C—N and C—C bonds lengths in the cation vary from 1.340 (3) to 1.383 (3) Å and from 1.346 (4) to 1.402 (3) Å, respectively. The C—N—C bond angles range from 117.8 (2) to 129.7 (2)° and the N—C—C angles range from 119.0 (2) to 123.4 (2)°. The dihedral angle between the C3–C7/N4 and C8–C12/N5 rings is 1.92 (13)°. These data are comparable to those found for other compounds containing dpa as an organic template (Bowes *et al.*, 2003 ▸; Willett, 1995 ▸). In the cation, the pyridyl nitro­gen atoms are arranged on both sides of the central N3 atom and assume a *cis* conformation (Fig. 1 ▸). The (C_10_H_10_N_3_)^+^ cation is monoprotonated at the pyridyl-N4 atom, which leads to the the formation of a short and presumably strong intra­molecular N4—H4*A*⋯N5 hydrogen bond (Table 1 ▸), which generates an *S*(6) ring (Fig. 2 ▸).

The Ni^2+^ ion of the anion is located on a crystallographic inversion center and coordinates four terminal (non-bridging) cyanide ligands, exhibiting a square-planar geometry. The bond lengths and angles in the anion are in good agreement with those found in other [Ni(CN)_4_]^2−^ salts (Paharová *et al.*, 2003 ▸; Karaağaç *et al.*, 2013 ▸).

## Supra­molecular features   

**Figure 3:**
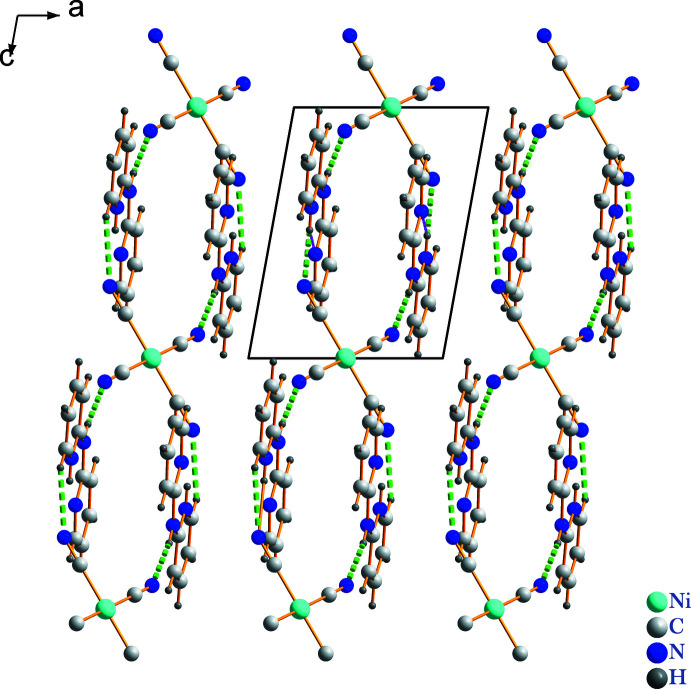
View parallel to the *ac* plane of the packing in (I) with hydrogen bonds shown as green dashed lines.

Fig. 3 ▸ shows the packing of (I) in a view along the *b-*axis direction, in which the organic and inorganic ions form chains propagating in the [101] direction linked by N—H⋯N and C—H⋯N hydrogen bonds. The pyridinium N4 atom in the cation, as well as forming the intra­molecular hydrogen bond described above, acts as donor to the cyanate N atom in the anion, in an N4—H4*A*⋯N1^ii^ [symmetry code: (ii) −*x* + 1, −*y* + 1, −*z* + 1) link (Table 1 ▸). The secondary amino group (N3*H*) forms a strong N3—H3*A*⋯N2 hydrogen bond with a cyano group acceptor and the H3*A*⋯N2 distance is 2.0 Å. Fig. 3 ▸ shows the parallel offset π-stacking contacts between pyridyl groups [centroid–centroid distance of 4.3421 (16) Å] and parallel face-centred π-stacking inter­actions between the *S*(6) centroids and pyridyl groups [centroid–centroid distance of 3.487 (2) Å].

## Hirshfeld surface analysis   

**Figure 4:**
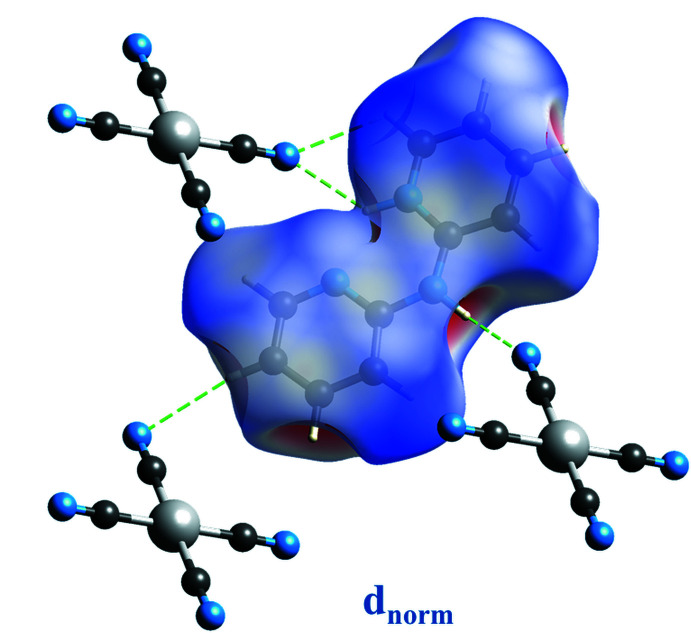
Hirshfeld surface of (I) mapped over *d*_norm_.

**Figure 5:**
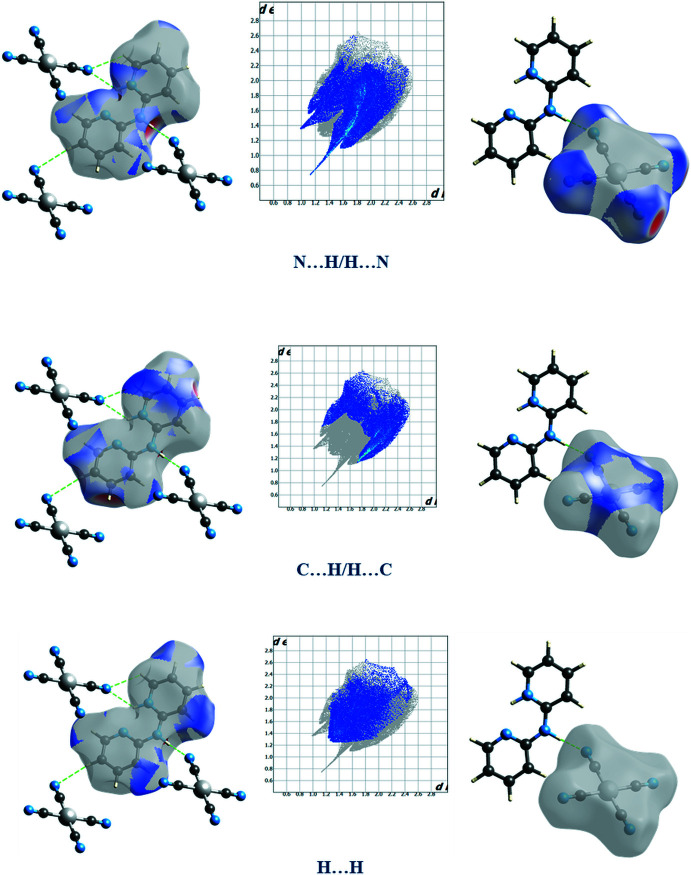
Two-dimensional fingerprint plots and relative contributions for (I) resolved into all, N⋯H, C⋯H and H⋯H contacts.

Hirshfeld surface calculations (Spackman & Jayatilaka, 2009 ▸) for (I) were performed in order to further characterize the supra­molecular association. The Hirshfeld surfaces and two-dimensional fingerprint plots (McKinnon *et al.*, 2007 ▸) calculated using *CrystalExplorer 17.5* (Turner *et al.*, 2017 ▸) are shown in Figs. 4 ▸ and 5 ▸, respectively. The red spots on the Hirshfeld surface represent strong inter­action through N—H⋯N and C—H⋯N hydrogen bonding, whereas the blue color represents a lack of inter­action. The presence of π–π stacking inter­actions is indicated by adjacent red and blue triangles on the shape-index surface (Fig. S1*a* in the supporting information). Areas on the Hirshfeld surface with high curvedness (Fig. S1*b*) can be related to the planar packing arrangement of the cations. The most abundant inter­molecular inter­actions in the crystal packing (Fig. 5 ▸) are N⋯H/H⋯N, C⋯H/H⋯C and H⋯H with percentage contributions 37.2, 28.3 and 21.9%, respectively. The presence of weak π–π stacking inter­actions between the cationic rings are reflected in the 4.6 and 3.8% contributions from C⋯C and C⋯N/N⋯C contacts to the Hirshfeld surfaces of the cations. The analysis reveals the lowest contribution of Ni⋯N (1.7%), Ni⋯C (1.3%) and N⋯N (1.2%) contacts.

## Database survey   

A search of the Cambridge Structural Database (Version 5.41, last update November, 2019; Groom *et al.*, 2016 ▸), for the tetra­cyano­nickelate moiety revealed 532 hits. Most of them are complexes of [Ni(CN)_4_]^2–^ anions with different metal–ligand coordination cations. Salts containing tetra­cyano­nickelate anions and organic cations corresponded to 38 hits.

A compound closely related to the title compound is (C_10_H_11_N_3_)·[CuCl_4_] (Willett, 1995 ▸; CSD refcode ZAMCEV), which crystallizes in the same space group of *P* 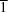. In this compound the cation is diprotonated and the pyridyl nitro­gen atoms are in a *cis* conformation and the pyridine rings are significantly twisted away from coplanarity. The tetra­chloro­cuprate anion takes on a squashed tetra­hedral geometry.

## Synthesis and crystallization   

The title compound was synthesized solvothermally under autogenous pressure using a mixture of iron(II) sulfate hepta­hydrate (28 mg, 0.10 mmol), 2,2′-di­pyridyl­amine (17 mg, 0.10 mmol) and potassium tetra­cyano­nickelate(II) (24 mg, 0.10 mmol) in mixed solvents of water/ethanol (3:1 *v*/*v*, 20 ml). The mixture was sealed in a Teflon-lined autoclave and held at 423 K for 3 d, and then cooled to room temperature at a rate of 10 K per hour (yield 27%). Pale-yellow plates of (I) suitable for single-crystal X-ray diffraction analysis were selected.

## Refinement   

Crystal data, data collection and structure refinement details are summarized in Table 2 ▸. All H atoms were positioned geometrically in idealized positions and constrained to ride on their parent atoms, with C—H = 0.93 or N—H = 0.86 Å, and with *U*_iso_(H) = 1.2*U*_eq_(C,N).

## Supplementary Material

Crystal structure: contains datablock(s) I. DOI: 10.1107/S205698902001419X/hb7948sup1.cif

Structure factors: contains datablock(s) I. DOI: 10.1107/S205698902001419X/hb7948Isup2.hkl

Figure S1 Hirshfeld surface of (C10H10N3)2[Ni(CN)4] mapped with shape index (a) and curvedness (b). DOI: 10.1107/S205698902001419X/hb7948sup3.tif

CCDC reference: 2040378

Additional supporting information:  crystallographic information; 3D view; checkCIF report

## Figures and Tables

**Table 1:** Hydrogen-bond geometry (Å, °)

*D*—H⋯*A*	*D*—H	H⋯*A*	*D*⋯*A*	*D*—H⋯*A*
N3—H3*A*⋯N2	0.86	2.00	2.853 (3)	172
N4—H4*A*⋯N5	0.86	1.97	2.629 (3)	132
N4—H4*A*⋯N1^ii^	0.86	2.41	3.055 (3)	132
C5—H5⋯N1^ii^	0.93	2.68	3.206 (4)	117

Symmetry code: (ii) 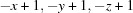.

**Table 2:** Experimental details

Crystal data	
Chemical formula	(C_10_H_10_N_3_)_2_[Ni(CN)_4_]
*M*_r_	507.21
Crystal system, space group	Triclinic, *P* 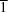
Temperature (K)	273
*a*, *b*, *c* (Å)	7.1046 (4), 9.1467 (4), 9.3833 (4)
α, β, γ (°)	100.182 (2), 98.729 (2), 97.444 (2)
*V* (Å^3^)	585.49 (5)
*Z*	1
Radiation type	Mo *K*α
μ (mm^−1^)	0.86
Crystal size (mm)	0.35 × 0.23 × 0.19

Data collection	
Diffractometer	Oxford Diffraction Xcalibur with Sapphire CCD detector
Absorption correction	Multi-scan (*CrysAlis RED*; Oxford Diffraction, 2009 ▸)
*T*_min_, *T*_max_	0.914, 0.962
No. of measured, independent and observed [*I* > 2σ(*I*)] reflections	16272, 3572, 2659
*R*_int_	0.052
(sin θ/λ)_max_ (Å^−1^)	0.715

Refinement	
*R*[*F* ^2^ > 2σ(*F* ^2^)], *wR*(*F* ^2^), *S*	0.048, 0.134, 1.07
No. of reflections	3572
No. of parameters	161
H-atom treatment	H-atom parameters constrained
Δρ_max_, Δρ_min_ (e Å^−3^)	1.01, −0.34

Computer programs: *CrysAlis CCD* and *CrysAlis RED* (Oxford Diffraction, 2009 ▸), *SHELXS97* (Sheldrick, 2015*a* ▸), *SHELXL2014/7* (Sheldrick, 2015*b* ▸), *DIAMOND* (Brandenburg, 2006 ▸) and *publCIF* (Westrip, 2010 ▸).

## supplementary crystallographic information

### Crystal data

**Table:**

(C_10_H_10_N_3_)_2_[Ni(CN)_4_]	*Z* = 1
*M**_r_* = 507.21	*F*(000) = 262
Triclinic, *P*1	*D*_x_ = 1.439 Mg m^−^^3^
*a* = 7.1046 (4) Å	Mo *K*α radiation, λ = 0.71073 Å
*b* = 9.1467 (4) Å	Cell parameters from 7173 reflections
*c* = 9.3833 (4) Å	θ = 2.8–27.9°
α = 100.182 (2)°	µ = 0.86 mm^−^^1^
β = 98.729 (2)°	*T* = 273 K
γ = 97.444 (2)°	Plate, pale yellow
*V* = 585.49 (5) Å^3^	0.35 × 0.23 × 0.19 mm

### Data collection

**Table:**

Oxford Diffraction Xcalibur Sapphire CCD detector diffractometer	2659 reflections with *I* > 2σ(*I*)
Radiation source: Enhance (Mo) X-ray Source	*R*_int_ = 0.052
ω scans	θ_max_ = 30.6°, θ_min_ = 2.2°
Absorption correction: multi-scan (CrysAlis RED; Oxford Diffraction, 2009)	*h* = −10→10
*T*_min_ = 0.914, *T*_max_ = 0.962	*k* = −13→13
16272 measured reflections	*l* = −13→13
3572 independent reflections	

### Refinement

**Table:**

Refinement on *F*^2^	Hydrogen site location: inferred from neighbouring sites
Least-squares matrix: full	H-atom parameters constrained
*R*[*F*^2^ > 2σ(*F*^2^)] = 0.048	*w* = 1/[σ^2^(*F*_o_^2^) + (0.0546*P*)^2^ + 0.3025*P*] where *P* = (*F*_o_^2^ + 2*F*_c_^2^)/3
*wR*(*F*^2^) = 0.134	(Δ/σ)_max_ < 0.001
*S* = 1.07	Δρ_max_ = 1.01 e Å^−^^3^
3572 reflections	Δρ_min_ = −0.34 e Å^−^^3^
161 parameters	Extinction correction: SHELXL-2014/7 (Sheldrick 2014, Fc^*^=kFc[1+0.001xFc^2^λ^3^/sin(2θ)]^-1/4^
0 restraints	Extinction coefficient: 0.091 (17)
Primary atom site location: structure-invariant direct methods	

### Special details

**Table:**

Geometry. All esds (except the esd in the dihedral angle between two l.s. planes) are estimated using the full covariance matrix. The cell esds are taken into account individually in the estimation of esds in distances, angles and torsion angles; correlations between esds in cell parameters are only used when they are defined by crystal symmetry. An approximate (isotropic) treatment of cell esds is used for estimating esds involving l.s. planes.

### Fractional atomic coordinates and isotropic or equivalent isotropic displacement parameters (Å^2^)

**Table:**

	*x*	*y*	*z*	*U*_iso_*/*U*_eq_	
Ni1	0.5000	0.0000	0.0000	0.04195 (19)	
C1	0.6743 (4)	0.0656 (3)	0.1772 (3)	0.0508 (6)	
N1	0.7800 (5)	0.1080 (3)	0.2861 (3)	0.0745 (8)	
C2	0.3639 (4)	0.1552 (3)	0.0568 (2)	0.0455 (5)	
N2	0.2831 (4)	0.2503 (3)	0.0942 (3)	0.0624 (6)	
N4	0.1820 (3)	0.7199 (2)	0.3981 (2)	0.0445 (4)	
H4A	0.1949	0.7110	0.4886	0.053*	
N5	0.2473 (3)	0.5418 (2)	0.5852 (2)	0.0484 (5)	
N3	0.2311 (3)	0.4715 (2)	0.3342 (2)	0.0492 (5)	
H3A	0.2354	0.3997	0.2625	0.059*	
C3	0.1977 (3)	0.6028 (3)	0.2949 (3)	0.0422 (5)	
C8	0.2592 (3)	0.4356 (3)	0.4722 (3)	0.0443 (5)	
C5	0.1462 (4)	0.8522 (3)	0.3626 (3)	0.0508 (6)	
H5	0.1351	0.9313	0.4365	0.061*	
C4	0.1807 (4)	0.6192 (3)	0.1479 (3)	0.0503 (6)	
H4	0.1942	0.5397	0.0753	0.060*	
C12	0.3276 (4)	0.2629 (3)	0.6262 (4)	0.0595 (7)	
H12	0.3538	0.1690	0.6407	0.071*	
C7	0.1442 (4)	0.7522 (3)	0.1118 (3)	0.0557 (6)	
H7	0.1314	0.7635	0.0144	0.067*	
C10	0.2763 (4)	0.5097 (3)	0.7197 (3)	0.0553 (6)	
H10	0.2695	0.5836	0.7998	0.066*	
C6	0.1264 (4)	0.8713 (3)	0.2224 (3)	0.0564 (7)	
H6	0.1013	0.9625	0.1994	0.068*	
C9	0.3006 (4)	0.2930 (3)	0.4869 (3)	0.0524 (6)	
H9	0.3096	0.2214	0.4055	0.063*	
C11	0.3157 (4)	0.3727 (4)	0.7447 (3)	0.0591 (7)	
H11	0.3341	0.3540	0.8397	0.071*	

### Atomic displacement parameters (Å^2^)

**Table:**

	*U*^11^	*U*^22^	*U*^33^	*U*^12^	*U*^13^	*U*^23^
Ni1	0.0617 (3)	0.0339 (2)	0.0330 (2)	0.01697 (18)	0.01155 (18)	0.00464 (15)
C1	0.0751 (18)	0.0380 (12)	0.0419 (12)	0.0238 (11)	0.0105 (12)	0.0040 (9)
N1	0.100 (2)	0.0626 (15)	0.0527 (14)	0.0317 (15)	−0.0091 (14)	−0.0053 (12)
C2	0.0628 (15)	0.0412 (12)	0.0328 (10)	0.0168 (11)	0.0073 (10)	0.0028 (9)
N2	0.0820 (17)	0.0554 (13)	0.0494 (12)	0.0333 (12)	0.0076 (12)	−0.0032 (10)
N4	0.0475 (11)	0.0447 (10)	0.0424 (10)	0.0101 (8)	0.0080 (8)	0.0096 (8)
N5	0.0520 (12)	0.0482 (11)	0.0468 (11)	0.0092 (9)	0.0106 (9)	0.0115 (9)
N3	0.0664 (14)	0.0403 (10)	0.0421 (10)	0.0166 (9)	0.0133 (10)	0.0022 (8)
C3	0.0382 (12)	0.0411 (11)	0.0485 (12)	0.0077 (9)	0.0070 (9)	0.0123 (9)
C8	0.0402 (12)	0.0451 (12)	0.0495 (13)	0.0045 (9)	0.0081 (10)	0.0160 (10)
C5	0.0544 (15)	0.0406 (12)	0.0582 (15)	0.0106 (10)	0.0111 (12)	0.0090 (11)
C4	0.0538 (15)	0.0525 (14)	0.0450 (13)	0.0137 (11)	0.0095 (11)	0.0069 (10)
C12	0.0574 (16)	0.0509 (15)	0.0743 (19)	0.0069 (12)	0.0059 (14)	0.0294 (14)
C7	0.0589 (16)	0.0636 (16)	0.0505 (14)	0.0141 (13)	0.0117 (12)	0.0228 (12)
C10	0.0572 (16)	0.0640 (16)	0.0456 (13)	0.0076 (13)	0.0120 (12)	0.0126 (12)
C6	0.0593 (16)	0.0494 (14)	0.0670 (17)	0.0147 (12)	0.0115 (13)	0.0242 (13)
C9	0.0585 (16)	0.0417 (12)	0.0576 (15)	0.0091 (11)	0.0097 (12)	0.0109 (11)
C11	0.0514 (15)	0.0747 (19)	0.0545 (15)	0.0034 (13)	0.0068 (12)	0.0291 (14)

### Geometric parameters (Å, º)

**Table:**

Ni1—C2	1.865 (2)	C8—C9	1.399 (3)
Ni1—C2^i^	1.865 (2)	C5—C6	1.346 (4)
Ni1—C1	1.867 (3)	C5—H5	0.9300
Ni1—C1^i^	1.867 (3)	C4—C7	1.365 (4)
C1—N1	1.145 (4)	C4—H4	0.9300
C2—N2	1.136 (3)	C12—C9	1.373 (4)
N4—C3	1.340 (3)	C12—C11	1.381 (4)
N4—C5	1.355 (3)	C12—H12	0.9300
N4—H4A	0.8600	C7—C6	1.399 (4)
N5—C8	1.326 (3)	C7—H7	0.9300
N5—C10	1.337 (3)	C10—C11	1.371 (4)
N3—C3	1.355 (3)	C10—H10	0.9300
N3—C8	1.383 (3)	C6—H6	0.9300
N3—H3A	0.8600	C9—H9	0.9300
C3—C4	1.402 (3)	C11—H11	0.9300
			
C2—Ni1—C2^i^	180.0	N4—C5—H5	119.4
C2—Ni1—C1	89.06 (10)	C7—C4—C3	119.8 (2)
C2^i^—Ni1—C1	90.94 (10)	C7—C4—H4	120.1
C2—Ni1—C1^i^	90.94 (10)	C3—C4—H4	120.1
C2^i^—Ni1—C1^i^	89.06 (10)	C9—C12—C11	119.7 (3)
C1—Ni1—C1^i^	180.0	C9—C12—H12	120.2
N1—C1—Ni1	178.8 (2)	C11—C12—H12	120.2
N2—C2—Ni1	178.6 (2)	C4—C7—C6	119.5 (2)
C3—N4—C5	121.3 (2)	C4—C7—H7	120.3
C3—N4—H4A	119.4	C6—C7—H7	120.3
C5—N4—H4A	119.4	N5—C10—C11	122.9 (3)
C8—N5—C10	117.8 (2)	N5—C10—H10	118.5
C3—N3—C8	129.7 (2)	C11—C10—H10	118.5
C3—N3—H3A	115.1	C5—C6—C7	119.1 (2)
C8—N3—H3A	115.1	C5—C6—H6	120.4
N4—C3—N3	119.7 (2)	C7—C6—H6	120.4
N4—C3—C4	119.0 (2)	C12—C9—C8	117.4 (3)
N3—C3—C4	121.3 (2)	C12—C9—H9	121.3
N5—C8—N3	117.0 (2)	C8—C9—H9	121.3
N5—C8—C9	123.4 (2)	C10—C11—C12	118.8 (3)
N3—C8—C9	119.6 (2)	C10—C11—H11	120.6
C6—C5—N4	121.3 (2)	C12—C11—H11	120.6
C6—C5—H5	119.4		

Symmetry code: (i) −*x*+1, −*y*, −*z*.

### Hydrogen-bond geometry (Å, º)

**Table:**

*D*—H···*A*	*D*—H	H···*A*	*D*···*A*	*D*—H···*A*
N3—H3*A*···N2	0.86	2.00	2.853 (3)	172
N4—H4*A*···N5	0.86	1.97	2.629 (3)	132
N4—H4*A*···N1^ii^	0.86	2.41	3.055 (3)	132
C5—H5···N1^ii^	0.93	2.68	3.206 (4)	117

Symmetry code: (ii) −*x*+1, −*y*+1, −*z*+1.
