# Supplementary figures and images for: Synthesis, crystal structure and Hirshfeld surface analysis of bis­{2-[(pyridin-2-yl)amino]­pyridinium} tetra­cyano­nickelate(II)
Source: Acta Crystallogr E Crystallogr Commun. 2020 Oct 30;76(Pt 11):1794–8. doi: 10.1107/S205698902001419X (PMC7643237; doi:10.1107/S205698902001419X)

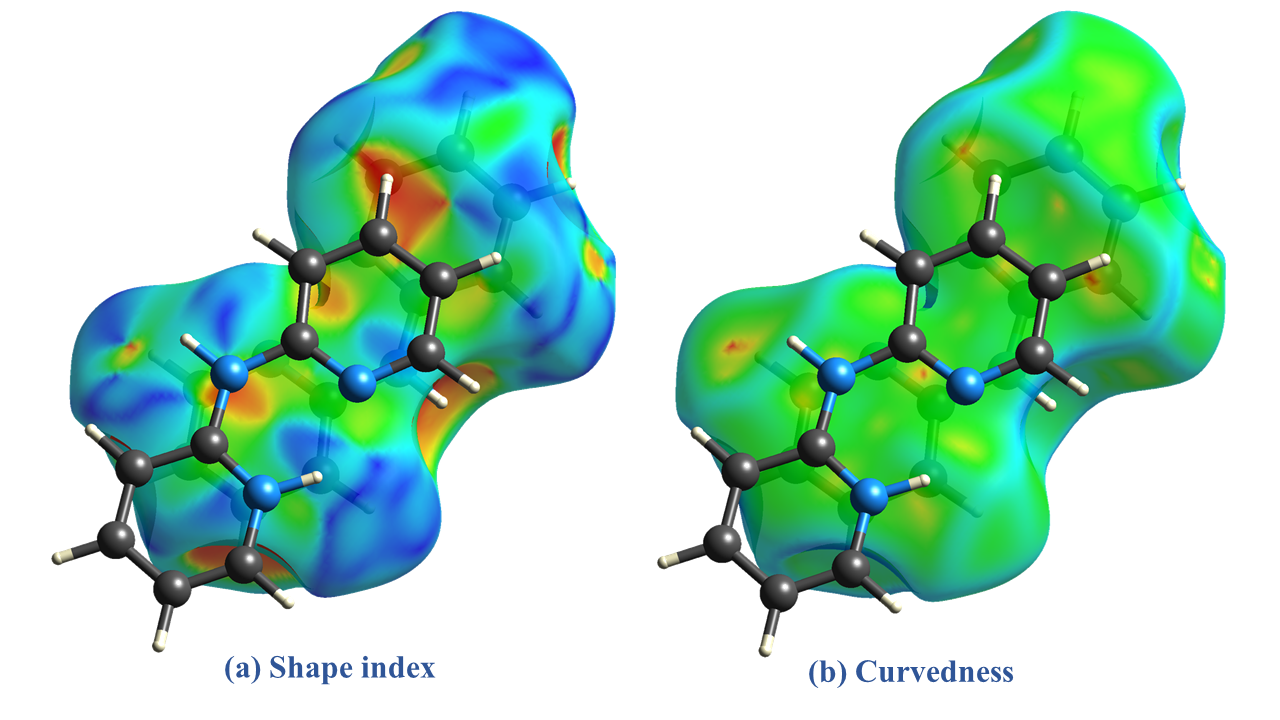

Supplement: Supplementary file 3 [file e-76-01794-sup3.tif]
